# Contrast Sensitivity of ON and OFF Human Retinal Pathways in Myopia

**DOI:** 10.1523/JNEUROSCI.1487-23.2023

**Published:** 2024-01-17

**Authors:** Sabina Poudel, Jianzhong Jin, Hamed Rahimi-Nasrabadi, Stephen Dellostritto, Mitchell W. Dul, Suresh Viswanathan, Jose-Manuel Alonso

**Affiliations:** Department of Biological and Visual Sciences, State University of New York College of Optometry, New York, New York 10036

**Keywords:** development, eye growth, luminance, pupil, retina, thalamus, visual cortex

## Abstract

The human visual cortex processes light and dark stimuli with ON and OFF pathways that are differently modulated by luminance contrast. We have previously demonstrated that ON cortical pathways have higher contrast sensitivity than OFF cortical pathways and the difference increases with luminance range (defined as the maximum minus minimum luminance in the scene). Here, we demonstrate that these ON–OFF cortical differences are already present in the human retina and that retinal responses measured with electroretinography are more affected by reductions in luminance range than cortical responses measured with electroencephalography. Moreover, we show that ON–OFF pathway differences measured with electroretinography become more pronounced in myopia, a visual disorder that elongates the eye and blurs vision at far distance. We find that, as the eye axial length increases across subjects, ON retinal pathways become less responsive, slower in response latency, less sensitive, and less effective and slower at driving pupil constriction. Based on these results, we conclude that myopia is associated with a deficit in ON pathway function that decreases the ability of the retina to process low contrast and regulate retinal illuminance in bright environments.

## Significance Statement

Contrast sensitivity is an important visual function that allows discriminating faint visual targets slightly lighter or darker than the background. We have previously demonstrated that ON and OFF cortical pathways signaling light and dark stimuli have different contrast sensitivity, and the difference increases with luminance range. Here, we demonstrate that these ON–OFF sensitivity differences are inherited from the retina and are affected by myopia (nearsightedness), a visual disorder that blurs vision at far distances and is becoming a world epidemic. We show that myopia is associated with a retinal deficit that makes ON pathways less effective at signaling contrast and regulating retinal illuminance. These results could have clinical implications and may lead to novel approaches for myopia control.

## Introduction

Light and dark visual stimuli are encoded by ON and OFF visual pathways that differ in their response modulations to luminance, contrast, and the spatiotemporal properties of the stimulus (Chichilnisky and Kalmar, 2002; Zaghloul et al., 2003; Jin et al., 2008, 2011; Komban et al., 2014; Kremkow et al., 2014; Rekauzke et al., 2016; Ravi et al., 2018; Rahimi-Nasrabadi et al., 2021; Williams et al., 2021; Harris and Dunn, 2023). In the primary visual cortex of cats and humans, ON pathways have higher contrast sensitivity and more pronounced contrast response saturation than OFF pathways, and the difference increases with luminance range (Kremkow et al., 2014; Pons et al., 2017; Rahimi-Nasrabadi et al., 2021). These ON–OFF differences in cortical function closely match the statistics of light and dark contrasts in nature (Ratliff et al., 2010; Cooper and Norcia, 2015; Rahimi-Nasrabadi et al., 2021) and the differences in human visual perception of light and dark stimuli (Chubb and Nam, 2000; Buchner and Baumgartner, 2007; Komban et al., 2011, 2014; Pons et al., 2017; Rahimi-Nasrabadi et al., 2023).

Previous studies indicate that ON visual pathways are affected by myopia, a visual disorder that blurs vision at far distance. Genetic mutations affecting ON pathways are associated with high myopia in humans (Dryja et al., 2005; Kurata et al., 2017; Al-Hujaili et al., 2019; Kim et al., 2021) and increased myopia progression in animal models (Pardue et al., 2008; Chakraborty et al., 2015). Moreover, the stimulation of dopaminergic receptors with apomorphine greatly reduces myopia progression in nonhuman primates, chicken, guinea pigs, and mice (Iuvone et al., 1991; Rohrer et al., 1993; Dong et al., 2011; Yan et al., 2015; Zhou et al., 2017), and because the only cells releasing dopamine in the retina are ON-pathway dopaminergic amacrine cells (Dacey, 1990; Munteanu et al., 2018), the activation of ON pathways should also reduce myopia progression. Myopia also decreases contrast sensitivity in humans (Jaworski et al., 2006; Stoimenova, 2007), as would be expected from weakened ON pathways that have higher contrast sensitivity than OFF pathways. Moreover, stimulus conditions that increase myopia progression such as optical blur, low light, and short viewing distance (Wallman and Winawer, 2004; Rose et al., 2008) also weaken the visual responses of ON more than OFF pathways (Kremkow et al., 2014; Pons et al., 2017; Jansen et al., 2019).

The function of ON and OFF human retinal pathways can be measured in humans noninvasively with electroretinography (Granit, 1933). The onset of flash stimuli presented on a rod-saturating background generates a negative wave (a wave) dominated by cone photoreceptors and modulated by OFF cone bipolar cells (Bush and Sieving, 1994), which is followed by a positive wave (b wave) dominated by ON bipolar cells (Dick and Miller, 1985; Gurevich and Slaughter, 1993). Conversely, the onset of dark stimuli generates a positive wave (d wave) dominated by OFF bipolar cells (Gurevich and Slaughter, 1993; Sieving et al., 1994). It is well known that myopia reduces the amplitude and increases the latency of the electroretinogram (Kawabata and Adachi-Usami, 1997; Westall et al., 2001; Hidajat et al., 2003; Wagner and Strasser, 2023). Stimuli driving myopia progression such as hyperopic defocus (Schaeffel et al., 1988) also reduce the electroretinogram amplitude (Ho et al., 2012; Chin et al., 2015; Khanal et al., 2019). However, it is currently unknown whether myopia differently affects ON and OFF retinal pathways in humans. To address this question, we measured the contrast response functions of ON and OFF pathways with electroretinography. Our findings demonstrate that, as in human visual cortex, contrast sensitivity is higher in ON than OFF retinal pathways, and the difference increases with luminance range. Moreover, we demonstrate that myopia severity is associated with neuronal deficits that make ON retinal pathways less responsive, slower, less sensitive, and less effective at driving pupil constriction. Taken together, these findings support the hypothesis that ON visual pathways are weaker in myopia.

## Materials and Methods

### Subjects

A total of 26 human subjects participated in the measurements of retinal responses with electroretinography and pupil responses with Tobii glasses: 6 emmetropes, 18 myopes, 1 amblyope, and 1 hyperope (Table 1). In addition, we measured the pupil response time-course with EyeLink 1000 (SR Research) in a separate group of 29 subjects (see below). Some subjects wore their contact lenses during the electroretinogram (ERG) recordings but most did not wear refractive correction to maximize their comfort during testing. Because the stimulus was a flash covering the entire visual field in a dome, refractive correction was not required. Differences in refraction and accommodation do not affect the strength of retinal responses to flash stimuli and have a limited effect on pupil size in bright backgrounds (average pupil diameter for corrected/uncorrected subjects, 2.36 ± 0.16/2.32 ± 0.19; *p* = 0.391; Wilcoxon test). The ERG was recorded with a device commercially available for clinical use (Diagnosys). Eye axial length was measured with a Lenstar 900 optical biometer (Haag-Streit). The study was approved by the institutional review board at State University of New York, College of Optometry, and followed the principles outlined in the Declaration of Helsinki. Informed consent was taken from each subject before the experiment.

**Table 1.** List of subjects

Subject	Sex	Age	Eye	Refraction (D)	Correction	Axial length (mm)
S1 (SV)	M	53	OD	Plano	No	23.18
			OS	Plano	No	23.27
S2 (SP)	F	25	OD	Plano	No	23.06
			OS	Plano	No	23.28
S3 (FO)	F	34	OD	Plano	No	24.00
			OS	Plano	No	23.90
S4 (AI)	M	23	OD	Plano	No	24.23
			OS	Plano	No	24.10
S5 (SN)	M	30	OD	Plano	No	23.65
			OS	Plano	No	23.41
S6 (DS)	F	31	OD	Plano	No	23.71
			OS	Plano	No	23.80
S7 (SD)	M	27	OD	−0.50/−1.25 × 170	No	24.54
			OS	−0.75/−1.00 × 020	No	24.32
S8 (RN)	M	29	OD	−1.50/−0.75 × 020	Contact lens	24.86
			OS	−1.25/−0.50 × 180	Contact lens	24.90
S9 (HRN)	M	26	OD	−2.50	No	25.36
			OS	−1.50	No	25.13
S10 (ML)	F	26	OD	−4.50	Contact lens	26.72
			OS	−3.25	Contact lens	26.33
S11 (LK)	F	26	OD	−4.75	No	25.34
			OS	−4.75	No	25.35
S12 (MM)	F	26	OD	−4.50/−0.75 × 169	No	22.98
			OS	−6.75/−1.25 × 170	No	24.24
S13 (EH)	F	30	OD	−5.00/−1.25 × 020	Contact lens	25.93
			OS	−4.50/−1.25 × 160	Contact lens	25.58
S14(JMA)	M	57	OD	−5.25/−0.75 × 060	No	27.88
			OS	−7.00/−0.75 × 090	No	28.03
S15 (JCN)	M	27	OD	−5.25/−1.25 × 160	No	26.30
			OS	−4.50/−1.75 × 015	No	25.91
S16 (TH)	F	23	OD	−6.25	Contact lens	27.12
			OS	−6.00	Contact lens	26.98
S17 (EM)	F	23	OD	−6.50	Contact lens	24.69
			OS	−6.50/−0.50 × 180	Contact lens	24.62
S18 (TT)	M	39	OD	−5.75/−2.25 × 015	No	25.66
			OS	−5.75/−2.25 × 158	No	25.81
S19 (CHO)	F	27	OD	−6.50	Contact lens	27.07
			OS	−7.00	Contact lens	27.17
S20 (ER)	F	25	OD	−7.00	Contact lens	26.72
			OS	−7.50	Contact lens	26.61
S21 (JS)	M	25	OD	−9.25	No	28.10
			OS	−9.50	No	28.18
S22 (MF)	F	24	OD	−10.25	No	26.81
			OS	−10.25	No	26.66
S23 (KC)	F	27	OD	−10.25/−0.25 × 150	Contact lens	27.83
			OS	−9.00/−0.75 × 055	Contact lens	27.44
S24 (BF)	M	23	OD	−9.00/−1.50 × 180	No	28.75
			OS	−11.25/−2.50 × 180	No	29.53
S25 (JJ)	M	44	OD	−0.75/−3.50 × 080 (a)	No	24.33
			OS	Plano	No	23.30
S26 (MS)	M	28	OD	+2.00	No	21.85
			OS	+2.25	No	21.82

From left to right, the table shows subject number and initials, sex (M, male; F, female), age in years, eye tested (OD, right eye; OS, left eye), refraction (spectacle prescription; a, amblyopic eye), type correction worn during the experiments (no, no correction), and eye axial length.

### Visual stimulation

Visual stimuli were generated in Matlab and imported as text files into a commercial device (Diagnosys) used to record the ERG. The visual stimuli were presented as sequences of flashes in a Ganzfeld dome (ColorDome; Espion system) to achieve full-field retinal stimulation. The background luminance was maintained at 500 cd/m^2^ for all stimulus conditions. We measured ERGs at two different luminance ranges (500 and 250 cd/m^2^) on two separate days. The 500 cd/m^2^ range was sampled at 50 cd/m^2^ luminance steps while varying the stimulus luminance between 500 and ∼0 cd/m^2^ for dark flashes or between 500 and 1,000 cd/m^2^ for light flashes. The 250 cd/m^2^ range was sampled at 25 cd/m^2^ luminance steps while varying the stimulus luminance between 500 and 250 cd/m^2^ for dark flashes or between 500 and 750 cd/m^2^ for light flashes. For each luminance range, the stimulus sequence was organized into two blocks of trials with the same luminance contrast values but different random order.

Each trial block was divided into two segments separated from each other by a 2.2 s resting period at the background luminance of 500 cd/m^2^. The first segment lasted 5.6 s and the second 5.2 s. All segments had flashes of 0.2 s preceded by 0.2 s of background luminance (500 cd/m^2^) and started with a period of adaptation also at background luminance (500 cd/m^2^). The adaptation period lasted 2 s for the first segment and 0.4 s for the second because the second segment was followed by a 2.2 s resting period already at background luminance. Each flash sequence was preceded by a red flash and terminated with a green flash, both lasting 0.4 s and having a luminance of 20 cd/m^2^. Subjects were instructed to stop blinking after seeing the red flash, start blinking after seeing the green flash, and use the periods between green and red flashes to rest the eyes and lubricate them by blinking. Each trial block was repeated 30 times and each luminance value was tested 60 times. The entire stimulus sequence including resting periods lasted around 20 min.

### ERG recordings

We used Dawson–Trick–Litzkow (DTL) electrodes to record the ERG signals from one or both eyes of each subject while allowing normal pupillary responses (the pupil was not pharmacologically dilated). Two reference electrodes (gold cups), one per eye, were placed at the right and left temple. A common ground electrode (also gold cup) was placed at the subject’s forehead. The ERG signals were sampled at 1,000 Hz and the impedances of all the electrodes kept at values lower than 10 kΩ (measured before the recordings started). After the electrode placement was completed, the subjects were placed in front of the Ganzfeld stimulation dome with their heads and chins stabilized with a headrest and a chinrest. The subjects were instructed to fixate on a small red dot at the center of the dome during the ERG recordings.

### Pupil recordings

After the ERG recordings finished, we measured the variations in pupil size during one stimulus sequence lasting 31.2 s with Tobii Pro Glasses 2 (Tobii Technology). The subjects were instructed to blink only in the resting periods between green and red flashes, as in the ERG recordings. The Tobii glasses sampled the pupil size at 100 Hz with a resolution of 240 × 960 pixels. The glasses were also equipped with a frontal camera to record the flash sequence with a resolution of 1,920 × 1,080 pixels at a rate of 25 frames per second. Before the pupil recordings started, the subjects were asked to fixate at the center of a circular target placed at a distance of 1 m to calibrate eye position with the Tobii eye-tracking software.

The recording box of the Tobii Pro Glasses 2 stored the pupil data in a removable secure digital (SD) card as a JavaScript Object Notation (JSON) file. It also stored movies of the flash sequence as mp4 files. We imported the JSON file in Matlab and extracted the pupil diameter (pd) from both eyes measured in millimeters. We then converted the movie of the flash sequence to an 8 bit grayscale using the “rgb2gray” Matlab function and calculated the mean pixel intensity of all movie frames. The timestamps of the Tobii Pro Glasses 2 recordings were synchronized with the stimulus sequence by temporally aligning the onset of the first flash in the movie with the onset of the first flash in the stimulus sequence. The segments with blinks were removed from the pupil measurements before calculating the mean and standard deviation of pupil sizes across the entire stimulus sequence. Because the stimulus sequence was exactly the same in the retinal and pupil recordings, the mean and standard deviations of pupil size should be the same as if they were simultaneously recorded. Performing simultaneous retinal and pupil recordings would require the subjects to wear the Tobii glasses next to the ERG electrodes, which would reduce the retinal peripheral stimulation (the glasses frame blocks the peripheral view and the face cannot be inside the Ganzfeld because the glasses do not fit within the Ganzfeld rim). Also, measuring the pupil responses separately from the retinal recordings was much more comfortable for the subjects.

We also measured the pupillary light response with EyeLink 1000 (SR Research) in 29 additional subjects, 17 myopes and 12 controls (age range, 20–27 years). The subjects with myopia wore contact lenses during the experiment to achieve 20/20 vision (spectacle prescription range, −2.00 to −10.5 diopters). The EyeLink 1000 allowed us to measure the pupillary light response with higher sampling frequency than the Tobii glasses (1,000 Hz vs 100 Hz) and quantify more accurately the response time-course. The pupillary light response was driven with visual stimuli generated with custom Matlab software and presented on a monitor (BenQ, 120 Hz) placed at a distance of 64 cm from the eye. The pupil recordings were collected and stored in a computer running Plexon (Plexon). Subjects used a chinrest/headrest to hold their head steady in front of the monitor and had a patch covering their left eye (all measurements were taken with the right eye). Each stimulus trial started with a blue fixation dot that turned red to signal the subjects that they had to keep their eyes open and restrain from blinking. After a subject fixated on the red dot for 1 s without blinking, two flashes of different luminance were presented, each flash lasting 25 ms and being followed by a black background lasting 3 s. The stimulus trial ended with a green dot that signaled the subjects the start of a resting period that could be used to close and lubricate the eyes for 2–3 s. The subjects had to fixate the red dot without blinking for the entire trial (7.05 s) and any blink or fixation break made the trial to be aborted and repeated. The subjects were tested with 36–48 trials, which allowed measuring the pupil response to each luminance 6–8 times. The light flashes were presented on a black background at 12 different luminance levels, ranging from 0.35 to 256 cd/m^2^ (log scale distribution, 0.35, 0.38, 0.43, 0.53, 0.73, 1.16, 2.15, 4.63, 11.28, 30.29, 87.62, 256 cd/m^2^). The room lights were turned off during the experiment. For each luminance level, we measured the amplitude of pupil constriction, the peak time, and the time at half-amplitude of pupil constriction. We then compared the values measured at all luminance levels between myopes and controls. Notice that the mean pupil sizes measured with EyeLink 1000 were around 1 mm larger than those measured with the Tobii glasses (3.41 ± 0.47 mm for EyeLink vs 2.3 ± 0.17 mm for Tobii glasses) because the visual stimulation was dimmer and weaker in the EyeLink than Tobii glasses recordings. When compared with the Tobii glasses, the EyeLink recordings were obtained with a background luminance 500 cd/m^2^ dimmer (500 cd/m^2^ for Tobii glasses vs ∼0 cd/m^2^ for EyeLink), a luminance range ∼250 cd/m^2^ lower (maximum target-background luminance, 500 cd/m^2^ for Tobii glasses vs 256 cd/m^2^ for EyeLink), and a flash duration almost one order of magnitude shorter (200 ms for Tobii glasses vs 25 ms for EyeLink).

### Data analysis

The ERG data was exported as a text file from the commercial ERG device (Diagnosys) and analyzed in Matlab. The exported ERG signals were converted to microvolts (µV) and passed through a low-pass filter with a cutoff frequency of 499 Hz. Then, the signals were passed through a high-pass filter with a cutoff frequency that varied across subjects between 0.1 and 9 Hz to make the recording baseline as flat as possible and eliminate slow baseline drifts. Trials with transients larger than ±1,000 µV due to blinks or movement artifacts were rejected and not included in the analysis (average across subjects, 3.33 ± 1.1 trials out of 30 trials in total). After the recording baseline was flat and the blink/movement artifacts rejected, we averaged the ERG responses to each luminance contrast within 0–0.2 s of the flash onset. Responses to the onset of a light flash were classified as ON responses, and responses to the onset of a dark flash were classified as OFF responses. ERG responses to the onset of a light flash had a negative wave (a wave) followed by a positive wave (b wave), whereas responses to the onset of a dark flash had a positive wave (d wave). The signal-to-noise ratio (SNR) was calculated separately for each luminance contrast as the ratio between the maximum absolute ERG voltage (within 40 ms of the flash onset) and the noise voltage level. The noise level was defined as the standard deviation of the ERG signal within 0.2 s preceding the flash onset, when the stimulation dome was at 500 cd/m^2^ background luminance. An SNR threshold was set for each subject and stimulus polarity to maximize the number of ON b waves and OFF d waves measured with 10 different contrasts (average SNR threshold across subjects, 2.5 ± 1.2 for ON and 2.9 ± 1.1 for OFF). ON b waves and OFF d waves that passed the SNR threshold were measured at the maximum value between 10 and 40 ms following the stimulus onset of a light flash (ON b wave) or dark flash (OFF d wave). ON a waves that passed the SNR threshold were measured at the minimum value of the ON ERG response between 0 and 40 ms following the stimulus onset. ERG waves (a, b, or d) that did not pass the SNR threshold (e.g., at the lowest contrasts) were measured at the time at which the nearest contrast generated an ERG response that passed the SNR threshold.

The response amplitude of ON b waves and OFF d waves were used to calculate the contrast response functions of the ON and OFF retinal pathways. We also measured the latencies of ON b waves and OFF d waves as the mean latency of responses to the four highest contrasts of light flashes (ON response latencies) and dark flashes (OFF response latencies). The contrast response functions were fitted with a Naka–Rushton function:

(1) $$R(C)=\mathrm{Baseline}+R_{\max}\frac{C^{n}}{C_{50}^{n}+\;C^{n}}\;$$

where *R*(*C*) is the retinal voltage response to luminance contrast *C*, Baseline is the baseline voltage, *R*_max_ is the voltage response at the maximum (100%) contrast, *n* is the exponent of the function, and *C*_50_ is the luminance contrast that generated 50% of the maximum response. We extracted the following parameters of the contrast response function: *C*_50_, *n*, *R*_max_, and goodness of fit (*R*^2^). The *C*_50_ was normalized (C50n) by the luminance range (defined as the maximum flash–background luminance) to measure changes in contrast sensitivity with retinal illuminance (Rahimi-Nasrabadi et al., 2021). Throughout the paper, we measured contrast as (FL − BL) / *R*, where FL is the flash luminance, BL is the background luminance, and *R* is luminance range (maximum FL–BL). This normalized Weber contrast (Rahimi-Nasrabadi et al., 2021) allows us to compare our measurements with those obtained in other species and brain structures including our own measurements in cat and human visual cortex (Rahimi-Nasrabadi et al., 2021). The normalized Weber contrast can be also converted into a luminance difference by multiplying the contrast percentage by the luminance range. For example, 50% contrast measured with a luminance range of 500 cd/m^2^ corresponds to a 250 cd/m^2^ flash–background luminance difference.

### Experimental design and statistical analysis

We tested the statistical significance of paired comparisons with two-tailed Wilcoxon tests using the Matlab function sign-rank (e.g., comparisons between ON and OFF responses measured with the same luminance range or across luminance ranges). The Matlab function rank sum was used to compare differences in the amplitude and time-course of pupil constriction between myopes and controls. Pearson’s correlation coefficients were calculated using Matlab function corrcoef. We also used bootstrapping to measure the correlation between the range of retinal illuminance and the parameters of ON and OFF contrast response functions. To perform bootstrapping, we selected subjects and eyes generating strong retinal responses (at least 7 µV to the strongest stimuli, dark high-contrast flashes), a criterion that was met by 7 subjects and 12 eyes measured both at 250 and 500 cd/m^2^ (12 eyes × 2 luminance ranges = 24 contrast response functions). To measure each contrast response function, we performed 1,000 iterations of bootstrap resampling with replacement. In each iteration, we calculated the average response of 25 trial blocks selected randomly out of 30 trial blocks available (each trial block was randomly selected 25 times from the same pool of 30 trial blocks). Because each luminance value was repeated twice in each trial block, 25 trial blocks provided 50 luminance values to calculate each data point in each contrast response function. The average contrast response functions for each subject and luminance range were calculated by selecting bootstrap iterations in which the response to both dark and light 100% contrast flashes passed the SNR threshold of each subject (average number of bootstrap iterations across subjects, 971 ± 90). In addition, when measuring ON and OFF contrast response functions separately, we selected only functions with goodness of fit (*R*^2^) ≥ 0.4. The contrast response functions obtained from bootstrapping were used to calculate the *C*_50_ and *R*_max_ parameters.

## Results

**Figure 1.**
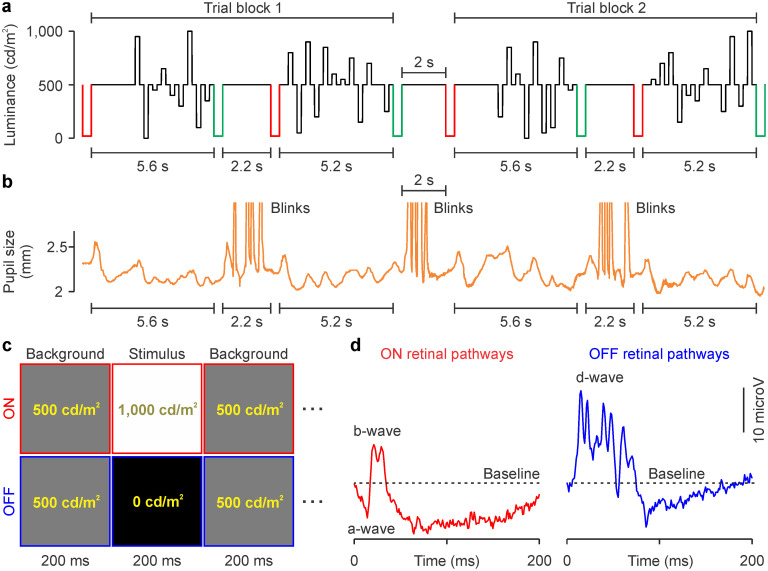
Stimulus protocol used to measure ON and OFF contrast response functions in human retina with flash electroretinography. ***a***, Stimulus sequence of light and dark flashes with a 500 cd/m^2^ luminance range (black traces). The same flash luminance values are presented in trial blocks 1 and 2 but in different random order. The red and green traces illustrate signals informing the subjects when to stop (red flash) and start blinking to lubricate the eyes (green flash). ***b***, Example measurements of pupil size during the stimulus sequence intercalated with blinks. ***c***, Example of a 600 ms fraction of the stimulus sequences used to measure responses from ON and OFF pathways. ***d***, Example retinal responses elicited by 100% contrast flashes illustrating a, b, and d waves (same traces as in Fig. 2*c*).

We used electroretinography to measure the contrast response functions of ON and OFF retinal pathways in humans. ON responses were driven by the onset of light flashes and OFF responses by the onset of dark flashes presented on a bright background of 500 cd/m^2^ (Fig. 1*a*; see Materials and Methods for details). The light and dark flashes also drove pupil responses (Fig. 1*b*) that were very restricted in amplitude because the background was bright (range of mean pupil diameter across subjects, 2–2.7 mm; range of standard deviations in pupil diameter across subjects, 0.05–0.14 mm). Red and green flashes were also intercalated with the stimulus sequence (Fig. 1*a*) to inform the subject when to blink (after a green flash) or not to blink (after a red flash). The same stimulus sequence (Fig. 1*c*) was used to measure ON and OFF contrast response functions (Fig. 1*d*) at two luminance ranges, 500 and 250 cd/m^2^, while keeping the background constant at 500 cd/m^2^. At the 500 cd/m^2^ range, the brightest and darkest flashes were 1,000 and ∼0 cd/m^2^ (500 cd/m^2^ brighter or darker than the background), whereas at the 250 cd/m^2^ range, the brightest and darkest flashes were 750 and 250 cd/m^2^ (250 cd/m^2^ brighter or darker than the background).

### Differences in contrast sensitivity between ON and OFF human retinal pathways

ON pathways have higher contrast sensitivity than OFF pathways in human visual cortex, cat visual cortex, macaque thalamus, cat thalamus, and the isolated retina of primates and guinea pigs (Chichilnisky and Kalmar, 2002; Zaghloul et al., 2003; Kremkow et al., 2014; Pons et al., 2017; Ravi et al., 2018; Archer et al., 2021; Rahimi-Nasrabadi et al., 2021). Therefore, we predicted that ON pathways should also have higher contrast sensitivity than OFF pathways in the human retina. Previous measurements of human electroretinography reported differences in contrast sensitivity between light and dark stimuli (Korth and Rix, 1984; Vukmanic et al., 2014). However, the interpretation of these differences was complicated by changes in temporal and luminance adaptation that affect ON–OFF pathway comparisons (Komban et al., 2014; Mazade et al., 2019; Rahimi-Nasrabadi et al., 2021; Mazade et al., 2022).

**Figure 2.**
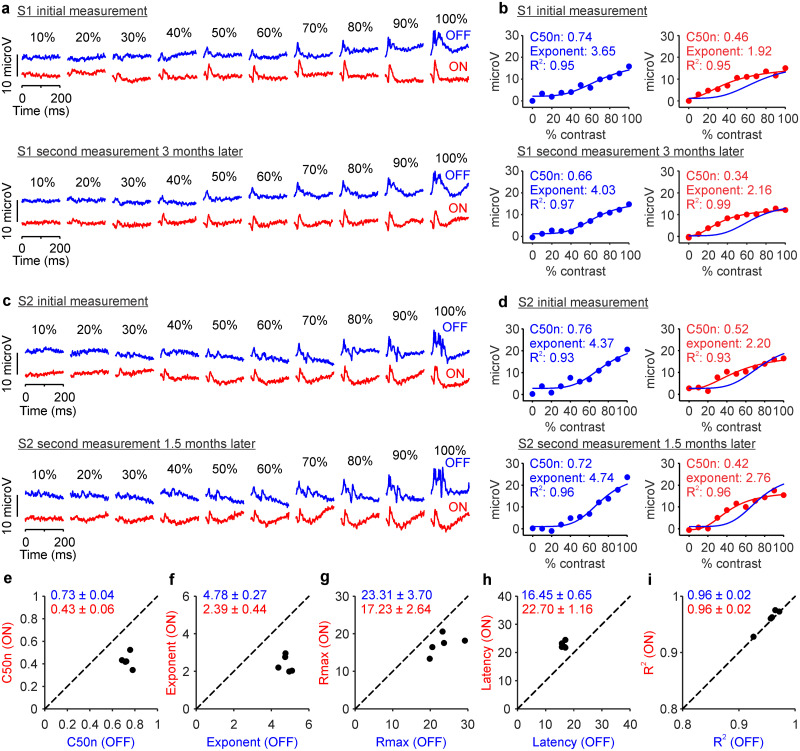
Contrast response functions of ON and OFF retinal pathways measured in the same individual subjects several months apart. ***a***, Top, ON (red) and OFF (blue) responses measured from an individual subject (S1) at 500 cd/m^2^ luminance range. Numbers at the top illustrate the flash temporal contrast. Bottom, Contrast responses from the same subject measured 3 months later. ***b***, Top left, OFF responses measured in subject S1 (blue circles) fitted with a Naka–Rushton function (blue line). The top left corner shows the values of C50n, exponent, and goodness of fit (*R*^2^). Top right, ON responses measured in subject S1 (red circles) fitted with a Naka–Rushton function (red line) with the fit for OFF responses overlaid (blue line). Bottom, Same as top for the contrast responses measured from subject S1 after 3 months. ***c***, ***d***, Same as ***a*** and ***b*** for a different subject (S2). ***e***, Scatterplot showing ON C50n and OFF C50n measured in five different days in subject S2. Numbers at the top left corner show means ± standard deviations. ***f***–***j***, Same as ***e*** for exponent (***f***), response at maximum contrast (***e***), latency (***h***), and goodness of fit (***j***).

In our experiments, we measured the responses of ON and OFF retinal pathways to the onset of light and dark flashes with 10 different temporal contrasts presented on a constant bright background. The ON and OFF contrast response functions measured with this approach (Fig. 2*a–d*) closely matched the ON and OFF contrast response functions measured in different animal models and brain structures (Chichilnisky and Kalmar, 2002; Zaghloul et al., 2003; Kremkow et al., 2014; Pons et al., 2017; Ravi et al., 2018; Archer et al., 2021; Rahimi-Nasrabadi et al., 2021). Moreover, the ON and OFF contrast response functions were well fit with the Naka-Rushton functions and could be accurately described with five parameters: the contrast generating half-maximum response normalized by the luminance range (C50n), the exponent of the function, the response to maximum contrast (*R*_max_), the response latency, and the goodness of fit (*R*^2^, Fig. 2*e–i*). All measurements and parameters extracted could be reliably replicated in recording sessions separated by several months in individual subjects (Fig. 2).

**Figure 3.**
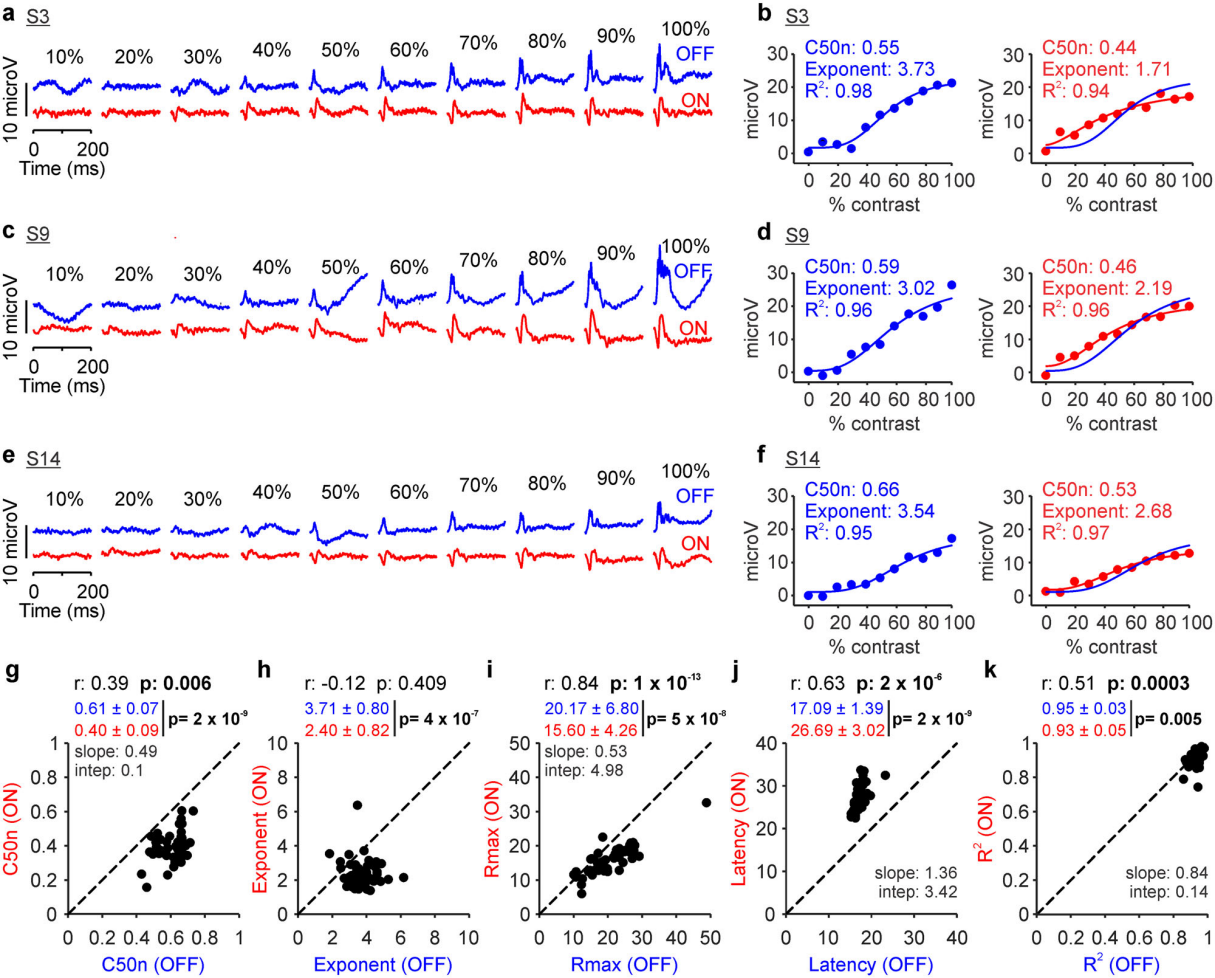
Retinal ON and OFF pathways have different contrast sensitivity when measured at 500 cd/m^2^ luminance range. ***a***, Retinal responses from subject S3 at luminance range 500 cd/m^2^. Same format as in Figure 1. ***b***, Contrast responses from subject S3 fitted with Naka–Rushton functions. ***c***–***f***, Same as ***a*** and ***b*** for subjects S9 (***c***, ***d***) and S14 (***e***, ***f***). ***g***, Scatterplot of C50n values from ON and OFF retinal pathways measured in 47 eyes from 26 human subjects. The top label reports Pearson’s correlation coefficient (*r*) next to the probability that the correlation is due to chance (*p*). The numbers below are means ± standard deviations for light (red) and dark stimuli (blue) next to the significance probability value (*p*) calculated with a two-sided Wilcoxon sign-rank test. The slope and intercept of a linear regression are reported below only for significant correlations (*p* values in bold). ***h***–***k***, Same as ***g*** for exponent (***h***), response at maximum contrast (***i***), latency (***j***), and goodness of fit (***k***).

Repeated measurements from the same individual subject at the 500 cd/m^2^ luminance range consistently demonstrated a higher contrast sensitivity (lower C50n), lower exponent, lower maximum response, and longer latency in ON than OFF retinal pathways (Fig. 2*e–i*). The same ON–OFF differences could be demonstrated in other individual subjects (Fig. 3*a–f*) and in 47 eyes from 26 subjects (only one eye was recorded in some subjects; see Materials and Methods). Notice that, in every eye tested, the contrast sensitivity was higher (C50n lower) and the response latency longer in ON than OFF retinal pathways (Fig. 3*g*,*j*). Moreover, in most eyes and subjects, the exponents and maximum responses were also lower in ON than OFF pathways (Fig. 3*h–i*). The contrast response functions were also slightly better fit in OFF than ON pathways (Fig. 3*k*).

All parameters of the contrast response function varied across eyes and the variations were highly correlated between the two pathways. The ON–OFF pathway correlations were significant for contrast sensitivity (Fig. 3*g*; *r*: 0.39; *p*: 0.006), response strength (Fig. 3*i*; *r*: 0.84; *p* < 0.0001), latency (Fig. 3*j*; *r*: 0.63; *p* < 0.0001), and goodness of fit (Fig. 3*k*; *r*: 0.51; *p* < 0.001). Across eyes and subjects, variations in contrast sensitivity and response latency were more pronounced in ON than those in OFF pathways (Fig. 3*g*,*j*), whereas variations in response strength were more pronounced in OFF than those in ON pathways (Fig. 3*i*). These results indicate that at 500 cd/m^2^ luminance range, ON retinal pathways have higher contrast sensitivity but generate weaker and slower responses than OFF retinal pathways. They also demonstrate that contrast sensitivity, response strength, and latency vary considerably across subjects, and the variations are correlated between ON and OFF pathways.

### Luminance range changes contrast response functions in the human retina

**Figure 4.**
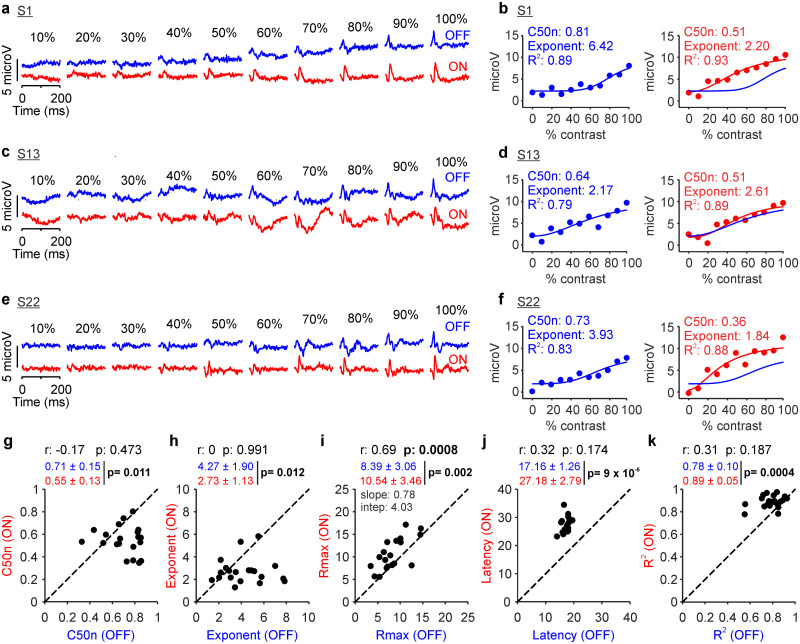
Retinal ON and OFF contrast response functions measured at 250 cd/m^2^ luminance range. The format is the same as Figure 2. ***a***, Example ERG response from subject S1 for luminance range 250 cd/m^2^. ***b***, Contrast responses from subject S1 fitted with a Naka–Rushton function. ***c***, Same as ***a*** for subject S13. ***d***, Same as ***b*** for subject S13. ***e***, Same as ***a*** for subject S22. ***f***, Same as ***b*** for subject S22. ***g***, Scatterplot showing ON C50n and OFF C50n measured from 13 human subjects (*n* = 20 eyes). ***h***, Same as ***g*** for exponent. ***i***, Same as ***g*** for response at maximum contrast. ***j***, Same as ***g*** for latency. ***k***, Same as ***g*** for goodness of fit.

The differences between the contrast response functions of ON and OFF pathways could be also demonstrated at a lower 250 cd/m^2^ luminance range (Fig. 4*a–f*). The lower luminance range reduced the strength of the visual responses and the accuracy of the Naka–Rushton fits (Fig. 4*i*,*k*). However, the C50n and exponent remained significantly larger and the response latency significantly shorter in OFF than those in ON pathways (Fig. 4*g–j*).

The higher contrast sensitivity of ON than OFF pathways was very consistent across subjects and could be demonstrated in both emmetropes and myopes. At 500 cd/m^2^ luminance range, the contrast sensitivity was higher (C50n lower) in every eye that we tested (Fig. 3*g*), including both emmetropes and myopes (Table 1). At the lower 250 cd/m^2^ luminance range, retinal responses became weaker and the ON–OFF difference in contrast sensitivity was also more variable. However, the average contrast sensitivity was still higher in ON than that in OFF pathways in both myopes and emmetropes (ON vs OFF, 0.56 ± 0.14 vs 0.77 ± 0.08; *n* = 11 eyes; *p* = 0.001 for myopes; 0.52 ± 0.12 vs 0.69 ± 0.14; *n* = 7 eyes; *p* = 0.097 for emmetropes), although the difference only reached significance in the larger sample of myopes.

**Figure 5.**
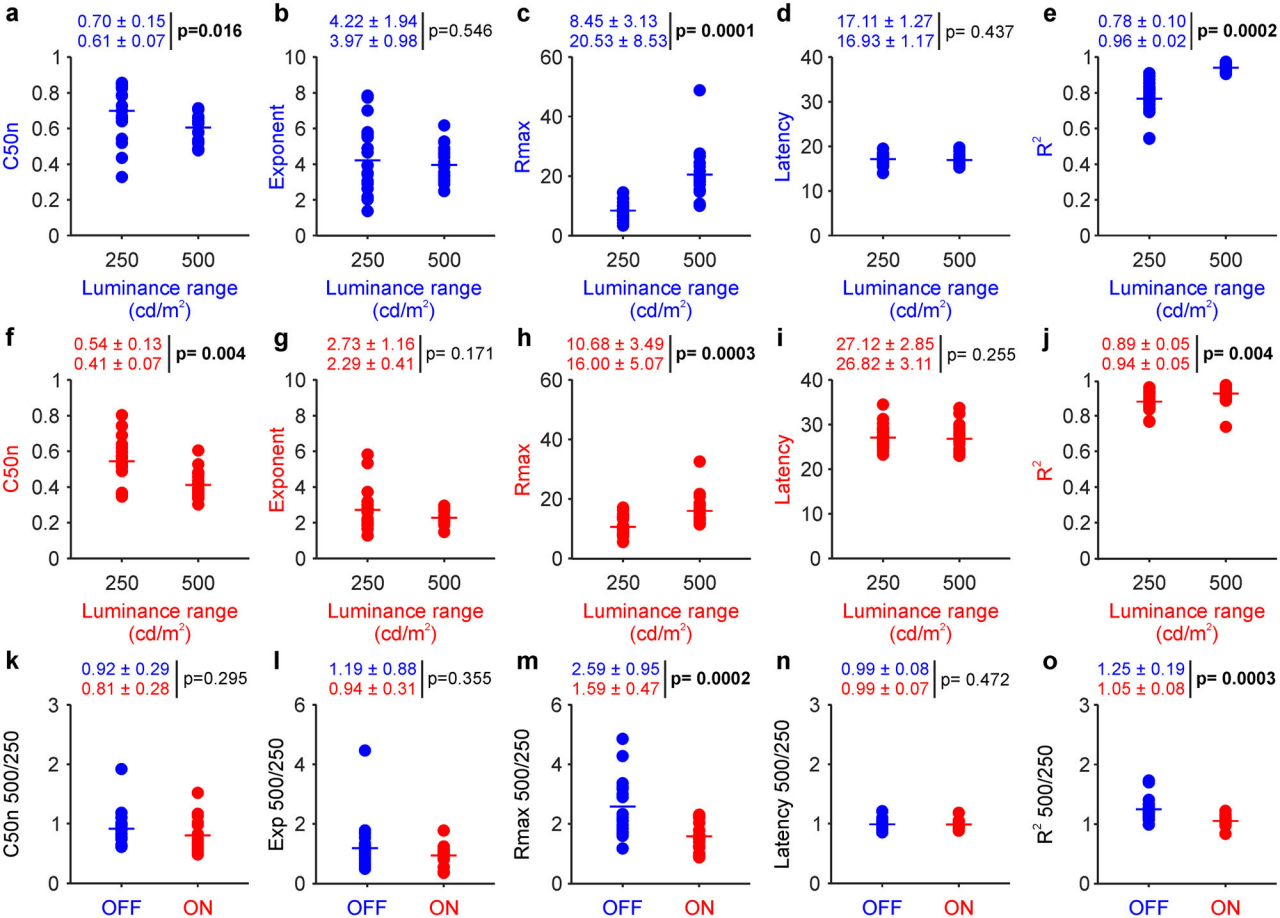
Expanding the luminance range from 250 to 500 cd/m^2^ increases contrast sensitivity and response strength in both ON and OFF retinal pathways. ***a***–***e***, Contrast sensitivity (C50n), exponent, *R*_max_, latency, and *R*^2^ of contrast response functions from OFF retinal pathways measured in 19 eyes from 13 subjects. The numbers at the top of each panel are average and standard deviations measured for each parameter at 250 cd/m^2^ (first number line) and 500 cd/m^2^ (second number line) luminance ranges; *p* is the probability that the parameter values are the same (Wilcoxon tests, significant *p* values highlighted in bold). ***f***–***j***, Same as ***a***–***e*** for ON pathways. ***k***–***o***, Same as ***a***–***e*** for 500/250 ratio comparisons between ON and OFF pathways. Ratios calculated by dividing parameters measured at 500 cd/m^2^ by those at 250 cd/m^2^.

The most dramatic effect of reducing the luminance range from 500 to 250 cd/m^2^ was an increase in the ON/OFF ratio of response strength. Decreasing the luminance range weakened the responses from OFF more than ON retinal pathways, making the ON/OFF response ratio larger (Fig. 4*a–f*; notice scale difference when comparing with Fig. 3*a–f*). Whereas at 500 cd/m^2^ luminance range, the average response was stronger in OFF than that in ON pathways (Fig. 3*i*; OFF/ON, 20.17 ± 6.8/15.6 ± 4.26 µV; *p* < 0.0001), at 250 cd/m^2^ luminance range, the average response was stronger in ON than that in OFF pathways (Fig. 4*i*; OFF/ON, 8.39 ± 3.06/10.54 ± 3.46 µV; *p* = 0.002). The response strengths of ON and OFF pathways remained significantly correlated at 250 cd/m^2^ luminance range (Fig. 4*i*; *r*: 0.69 *p* < 0.001), but the correlations for contrast sensitivity and response latency did not reach significance (Fig. 4*g*,*j*; *r* = −0.17; *p* = 0.473 for C50n; *r* = 0.32; *p* = 0.174 for latency). Decreasing the luminance range from 500 to 250 cd/m^2^ also reduced the contrast sensitivity, maximum response, and goodness of fit in both ON and OFF retinal pathways (Fig. 5*a*,*c*,*e* for OFF and *f*,*h*,*j* for ON pathways) but did not cause significant changes in the exponent and response latency (Fig. 5*b*,*d* for OFF and *g*,*i* for ON pathways). The reduction in response strength and goodness of fit was more pronounced in OFF than that in ON pathways (Fig. 5*m,o*), but there were no significant differences in other parameters (Fig. 5*k*,*l*,*n*). These results indicate that reducing the luminance range from 500 to 250 cd/m^2^ keeps contrast sensitivity higher and response latency longer in ON than OFF retinal pathways but weakens the responses of OFF more than ON retinal pathways and decreases the correlations between pathways.

**Figure 6.**
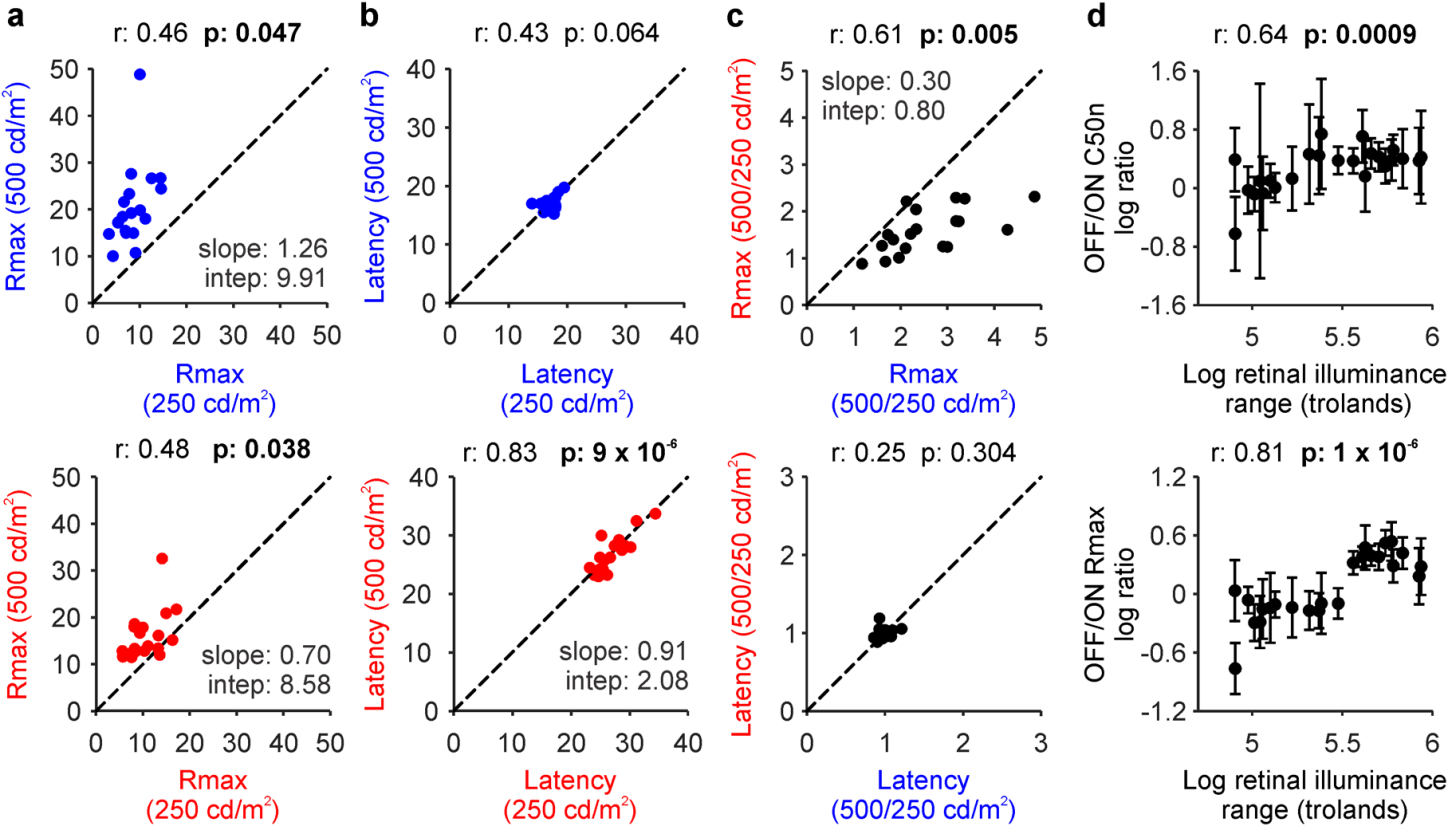
Luminance range increases response strength in OFF more than ON pathways and contrast sensitivity in ON more than OFF pathways. ***a***, Correlation between responses at maximum contrast (*R*_max_) measured at 250 and 500 cd/m^2^ luminance ranges in OFF (top) and ON pathways (bottom). ***b***, Same as a for response latency (***a***–***c***, 19 eyes, 13 subjects). ***c***, Correlation between ON (red) and OFF pathways (blue) for the *R*_max_ (top) and latency (bottom) 500/250 cd/m^2^ ratios. ***d***, Correlations in logarithmic scales between retinal illuminance and OFF/ON ratios of contrast sensitivity (top) and *R*_max_ (bottom), calculated by bootstrapping (see Materials and Methods; 12 eyes, 6 subjects).

When compared with our previous measurements in human visual cortex (Rahimi-Nasrabadi et al., 2021), decreasing the luminance range from 500 to 250 cd/m^2^ weakened the visual responses in human retina twice as much as in the visual cortex (ON + OFF pathway average 500/250 cd/m^2^; 18.27 ± 7.29 µV/9.57 ± 3.46 µV; *p* = 1.2 × 10^−7^ in the retina; 3.29 ± 0.91 µV/2.94 ± 1.11 µV; *p* < 3.5 × 10^−48^ in cortex; cortical measurements from Rahimi-Nasrabadi et al., 2021). The strengths of the retinal responses measured at 500 and 250 cd/m^2^ luminance ranges were weakly correlated across subjects in both ON and OFF pathways (Fig. 6*a*) whereas the response latencies were strongly correlated only in ON pathways (Fig. 6*b*). The response ratio between the two luminance ranges (*R*_max_ 500/250) was also correlated between pathways, but the latency ratio was not (Fig. 6*c*). These results indicate that increasing the luminance range strengthens the responses of OFF more than ON retinal pathways (Fig. 6*a*) but does not change the ON–OFF latency differences (Fig. 6*b*).

Our electroencephalography measurements in human visual cortex also demonstrate that differences in contrast sensitivity between ON and OFF pathways increase with retinal illuminance (Rahimi-Nasrabadi et al., 2021). We could not replicate this finding in the human retina by simply comparing the mean ratios of contrast sensitivity (Fig. 5*k*) because at the lower 250 cd/m^2^ luminance range, retinal responses were weak and the functional fits less accurate than at the 500 cd/m^2^ range. Therefore, to increase the accuracy of the comparison between retina and cortex, we analyzed the retinal measurements with the same bootstrapping method used to analyze the cortical data (Rahimi-Nasrabadi et al., 2021).

The bootstrapping analysis revealed a significant correlation between retinal illuminance and the OFF/ON ratio of contrast sensitivity in human retina (Fig. 6*d*; *r* = 0.64; *p* = 0.0009; bootstrap analysis), replicating our findings in human visual cortex (compare Fig. 6*d* with Fig. 5*f* of Rahimi-Nasrabadi et al., 2021). As in the visual cortex, increasing the retinal illuminance made the OFF/ON ratio of C50n larger. Moreover, when ON and OFF pathways were analyzed separately, the retinal illuminance was significantly correlated with contrast sensitivity in ON but not OFF pathways (*r* = −0.783; *p* = 0.000006 for ON; *r* = 0.056; *p* = 0.796 for OFF; bootstrap analysis). This result indicates that, as retinal illuminance increases, the C50n remains relatively constant in OFF pathways but decreases in ON pathways until it reaches a minimum that saturates the OFF/ON C50n ratio (Fig. 6*d*). We also found a significant correlation between retinal illuminance and the OFF/ON ratio of response strength (Fig. 6*d*; *r* = 0.81; *p* = 1 × 10^−6^). Based on these results, we conclude that retinal illuminance increases contrast sensitivity in ON more than OFF retinal pathways while increasing the strength of retinal responses in OFF more than ON pathways.

**Figure 7.**
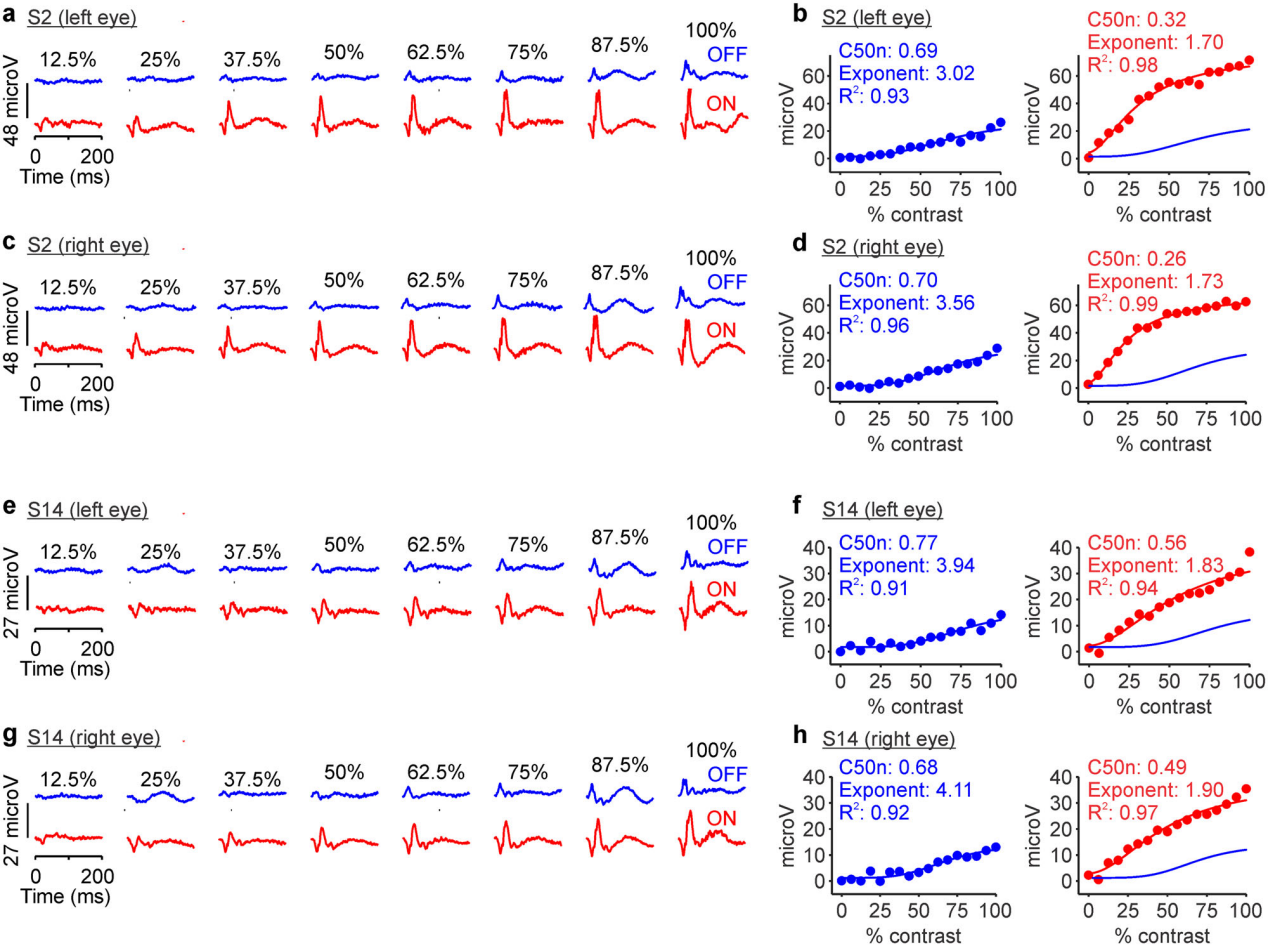
Retinal ON and OFF contrast response functions measured at 800 cd/m^2^ luminance range in different background luminance. The format is the same as Figure 2. ***a***, Example ERG responses from the left eye of subject S2 measured with a background luminance of 900 cd/m^2^ for dark stimuli and 100 cd/m^2^ for light stimuli. ***b***, OFF and ON contrast responses from the left eye of subject S2 fitted with a Naka–Rushton function. ***c***, ***d***, Same as ***a***,***b*** for the right eye of subject S2. ***e***–***h***, Same as ***a***–***d*** for subject S14.

Changes in background luminance also affected differently retinal and cortical responses. Cortical responses increased with luminance range but were not affected by changes in background luminance if the luminance range was kept constant (Rahimi-Nasrabadi et al., 2021). Conversely, retinal responses measured with flash electroretinography changed with both luminance range and background luminance. Changes in background luminance also affected the response ratio between ON and OFF pathways in the retina more than the cortex. For example, at a luminance range of 800 cd/m^2^, ON pathway responses to 900 cd/m^2^ bright stimuli presented on a 100 cd/m^2^ background were 2.5 times stronger than OFF pathway responses to 100 cd/m^2^ dark stimuli presented on a 900 cd/m^2^ background (Fig. 7). By comparison, in cat visual cortex, OFF pathway responses to a dark stimulus of 200 cd/m^2^ presented on a bright background of 800 cd/m^2^ were nearly identical in strength to ON pathway responses to a bright stimulus of 800 cd/m^2^ presented on a dark background of 200 cd/m^2^ (Supplementary Fig. 2*i* in Rahimi-Nasrabadi et al., 2021). It is important to notice that retinal responses measured with flash electroretinography are strongly dominated by retinal interneurons (presynaptic to retinal ganglion cells) that have very different properties than cortical cells. Responses from these retinal interneurons are likely to play an important role in regulating eye growth (Troilo et al., 1987; Norton et al., 1994), which makes the electroretinogram a helpful tool to investigate visual disorders such as myopia.

### ON retinal pathways are weaker and slower in myopia

**Figure 8.**
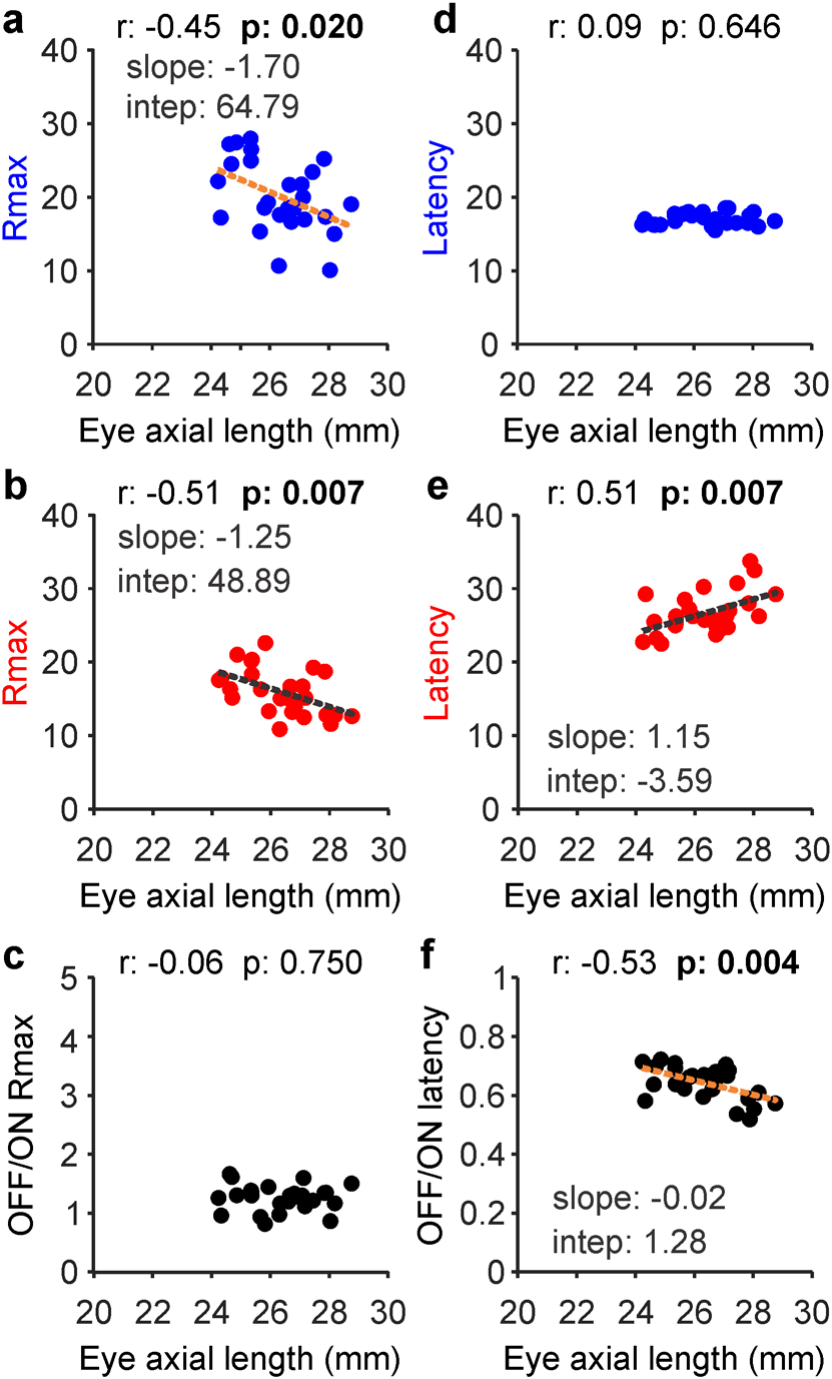
Myopia makes responses weaker in both ON and OFF pathways and slower in ON pathways. ***a***–***c***, Correlation between eye axial length and response strength (*R*_max_) for OFF (***a***), ON (***b***), and the OFF/ON pathway ratio (***c***). ***d***–***f***, Same as ***a–c*** for response latency. Same panel format as in Figure 6. Linear regressions (dotted lines) are shown only for significant correlations highlighted in bold.

In our electroretinography measures, the response strength and latency of ON and OFF retinal pathways were affected by myopia, a visual disorder that makes the eye grow longer. As eyes increased in axial length across subjects, retinal responses became weaker in both OFF (Fig. 8*a*; *r* = −0.45; *p* = 0.020) and ON visual pathways (Fig. 8*b*; *r* = −0.51; *p* = 0.007), keeping the OFF/ON ratio of response strength roughly constant (Fig. 8*c*; *r* = −0.06; *p* = 0.750). However, increases in eye axial length were significantly correlated with an increase of response latency in ON (Fig. 8*e*; *r* = 0.51; *p* = 0.007) but not OFF pathways (Fig. 8*d*; *r* = 0.09; *p* = 0.646), making the OFF/ON latency ratio decrease (Fig. 8*f*; *r* = −0.53; *p* = 0.004). These results indicate that, in myopia, visual responses are weaker in both ON and OFF pathways but slower only in ON pathways.

**Figure 9.**
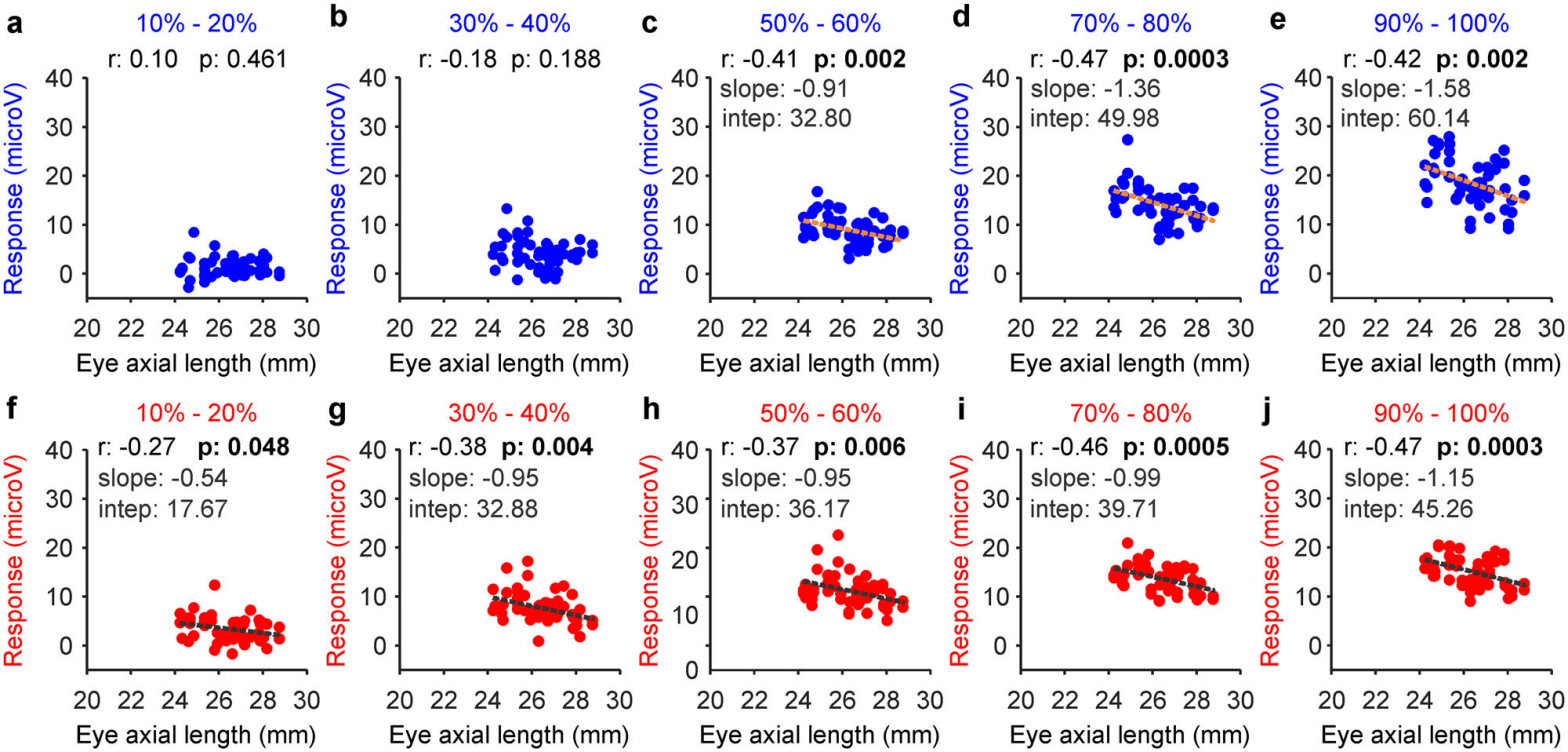
Myopia decreases responsiveness to mid-contrast in ON pathways and high contrast in both ON and OFF pathways. ***a***–***e***, Correlation between eye axial length and response strength in OFF retinal pathways measured at different contrast ranges (percentages at the top). ***f***–***j***, Same for ON pathways. In all panels, the numbers at the top report the correlation coefficient (*r*) and probability that the correlation is due to chance (*p*). The slope and intercept of the linear regressions (dotted lines) are reported only for significant correlations highlighted in bold.

Myopia also affected the contrast sensitivity of ON and OFF pathways differently. At low and medium contrasts (10–40%), the increase in eye axial length across subjects was significantly correlated with a response reduction in ON but not OFF pathways (Fig. 9*a*,*b*,*f–g*). Only at the highest contrast (50–100%), the increase in eye axial length was correlated with a response reduction in both pathways (Fig. *9c–e*,*h–j*; the correlations with the OFF/ON response ratio did not reach significance at any contrast). These results demonstrate that increases in eye axial length are associated with a response reduction to mid-contrast stimuli in ON pathways and to high contrast in both pathways.

### Pupil constriction is weaker and slower in humans with myopia

**Figure 10.**
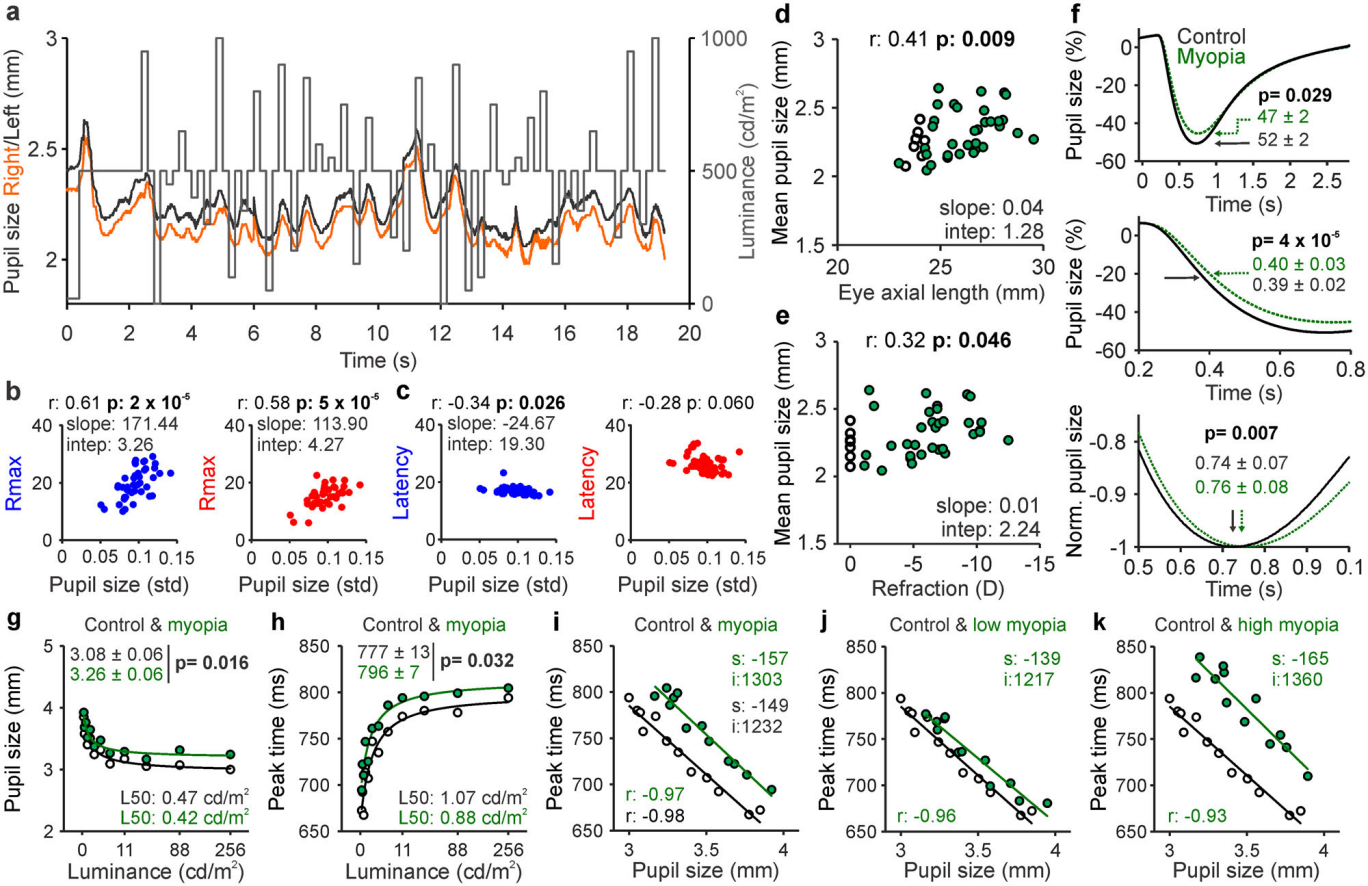
Pupil constriction becomes weaker and slower in myopia. ***a***, Pupil size measurements (right eye, orange; left eye, black) with Tobii glasses from an example subject in response to the electroretinogram flash sequence (gray), after subtracting the blink periods (∼10 s). ***b***, Correlation between the variation in pupil size during the sequence (std, standard deviation) and response strength (*R*_max_) for dark stimuli (blue) and light stimuli (red). ***c***, Same as ***b*** for latency. ***d***, Correlation between eye axial length and mean pupil size (emmetropes, open circles; myopes, filled green circles, 33 eyes; age range, 23–57 years old). ***e***, Correlation between eye refraction (*D*, diopters) and mean pupil size. Text labels in panels ***b–e*** report correlation coefficient (*r*), probability that the correlation is caused by chance (*p*), and slope and intercept of the linear regression. ***f***, Measurements with EyeLink 1000 demonstrating significant differences between myopes (green) and controls (black) in the mean percentage of pupil size change (top), time at half-amplitude of pupil constriction (middle), and peak time (bottom). Text labels report averages ± standard deviations and *p* value obtained by Wilcoxon tests in 12 emmetropes and 17 myopes (age range, 20–27 years old). ***g***, Mean pupil size at peak constriction measured at each luminance level and fit with a Naka–Rushton function. L50 is the luminance generating half of maximum pupil constriction. ***h***, Same as ***g*** for peak time. ***i***, Correlation between peak time and pupil size at peak constriction measured at each luminance level for controls versus myopes. *s* is the slope and *i* is the intercept of linear regression. ***j***, Same as *i* for control versus low myopia (<6 D). ***k***, Same as *i* for control versus high myopia (>6 D).

Retinal illumination is continuously adjusted by pupil constriction, which is driven by ON visual pathways in mammals (Beier et al., 2022). Therefore, if ON pathways are weaker and slower in myopia, pupil constriction should be also weaker and slower. We tested this prediction by measuring the pupil responses with the same flash sequence used in the electroretinogram recordings (Fig. 10*a*). Because the pupil measurements were obtained with high background luminance (500 cd/m^2^), both the mean and standard deviation of the pupil diameter were small (mean ± SD for the example left and right eyes from Fig. 10*a*: 2.24 ± 0.10 and 2.16 ± 0.10) and the differences between eyes were even smaller (Fig. 10*a*, black and orange lines; see also Poudel et al., 2023). Across subjects, the strength of the retinal response was not correlated with the mean pupil size (*r* = 0.08; *p* = 0.63 in OFF pathways; *r* = 0.03; *p* = 0.87 in ON pathways), but it was strongly correlated with its standard deviation (Fig. 10*b*; the standard deviation of pupil size was also weakly correlated with response latency in OFF pathways, Fig. 10*c*). These results indicate that retinal responses are strongest and fastest in subjects with the most responsive pupils (largest standard deviation), but not in those with the largest pupils (largest mean).

Previous studies reported larger pupils in myopes than emmetropes under low mesopic light of <5 cd/m^2^ (Cakmak et al., 2010; Linke et al., 2012; Guillon et al., 2016). However, under brighter light (>5 cd/m^2^), multiple studies found no significant differences (Jones, 1990; Winn et al., 1994; Charman and Radhakrishnan, 2009; Linke et al., 2012; Orr et al., 2015). In contrast to this previous work, our measurements demonstrate larger pupil sizes in myopes than emmetropes under bright light. Moreover, they demonstrate that pupil size is significantly correlated with both eye axial length (Fig. 10*d*) and optical refraction (Fig. 10*e*). In our sample of eyes (*n* = 40), the significance of the correlation was higher for eye axial length (*r* = 0.41; *p* = 0.009) than that for optical refraction (*r* = 0.32; *p* = 0.046) because one subject with low myopia (−1.875 and −1.50 diopters) had larger pupils than the average (2.53 and 2.64 mm at 500 cd/m^2^ background). However, removing this subject made the correlation for optical refraction just as strong (*r* = 0.46; *p* = 0.004) as for eye axial length.

Our measurements also demonstrate that pupil constriction is slower in myopes than that in emmetropes. To quantify the time-course of the pupil response, we performed measurements in a different group of subjects with EyeLink 1000, which has a higher sampling rate and allows more accurate stimulus–pupil temporal synchronization than Tobii glasses. These measurements demonstrate that pupil constriction is weaker (Fig. 10*f*, top) and slower (Fig. 10*f*, middle and bottom) in myopes than that in emmetropes. Therefore, we conclude that myopia makes pupil constriction weaker and slower but preserves its response variation (standard deviation) to diverse luminance transients.

These results strongly suggest that weak pupil responses in myopia subjects are a consequence of weak ON pathway signals that affect the sensory (and perhaps motor) components of the pupil reflex. However, the pupil could be also larger in myopia because longer eyes make the stimulus luminance dimmer (Quigley et al., 2018). Analyses of pupil response at different luminance values with EyeLink 1000 recordings makes this alternative explanation unlikely. Whereas a reduction of stimulus luminance should cause an increase in pupil size along the function relating both values (luminance and pupil size), a deficit in ON pathway function should have a multiplicative effect on the function. Our results are very consistent with a multiplicative effect (Fig. 10*g*). A reduction of stimulus luminance should also cause a reduction in the peak time of pupil constriction along the function relating both values, whereas an ON pathway deficit should cause a multiplicative effect. Our results are again consistent with a multiplicative effect (Fig. 10*h*). Finally, because the peak time and magnitude of pupil constriction are linearly related (the peak time increases with constriction amplitude), a reduction in stimulus luminance should reduce both the time and amplitude of the pupil constriction without changing their linear relation (same slope and/or intercept). Conversely, an ON pathway deficit should change the linear function. Once again, our results demonstrate that myopia causes a pronounced change in the linear function relating the amplitude and peak time of pupil constriction (Fig. 10*i*) and the change increases with myopia severity (Fig. 10*j,k*). Based on these results, we conclude that myopia makes pupil constriction weaker and slower because of a deficit in ON pathway function and not a reduction in stimulus luminance.

### Mechanism generating ON pathway deficits in myopia

**Figure 11.**
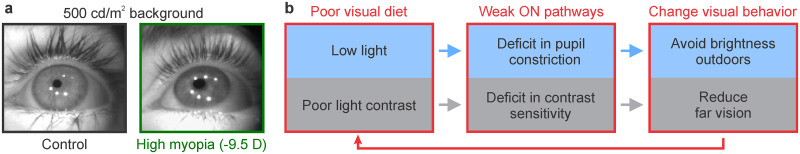
Proposed mechanism generating ON pathway deficits in myopia. ***a***, Eye images from example subjects with normal vision (control) and high myopia (−9.5 diopters). ***b***, Proposed mechanism generating ON pathway deficits through exposure to environments dominated by low light (blue) and low contrast (gray), such as reading for prolonged periods of time at low light. Environments with low light and poor light contrast (left) stimulate ON pathways poorly and may cause ON pathway deficits in pupil constriction and contrast sensitivity (middle). In turn, these visual deficits could make subjects avoid brightness outdoors (deficit in pupil constriction) and far vision (contrast decreases with viewing distance and the loss of contrast sensitivity makes far targets less visible). In turn, these changes in visual behavior (right) could make subjects spend more time indoors in environments dominated by low light and low contrast (red arrow).

We have previously proposed that poor stimulation of ON pathways in environments with low light and/or poor light contrast (e.g., reading under dim light) could make ON pathways weaker and reduce the ON pathway signals needed to stop eye growth (Poudel et al., 2023). We now directly demonstrate that ON pathways are weaker in human myopia and that ON pathway deficits are associated with a loss of contrast sensitivity and a deficit in pupil constriction (Fig. 11*a*). We notice that these visual deficits could potentially promote visual behaviors that increase the risk of myopia progression. For example, the weaker pupil response could lead to behaviors that avoid exposure to bright sky surfaces outdoors. Moreover, the loss of contrast sensitivity could lead to behaviors that maximize contrast by increasing near vision, as image contrast decreases with viewing distance (O’Shea et al., 1994). Near work and lack of outdoor activity are known to be major risk factors in myopia (Mutti et al., 2002; Rose et al., 2008; French et al., 2013; Wu et al., 2020). Therefore, by promoting these behaviors, the ON pathway deficits that we demonstrate could create a pathological cycle that increases myopia progression (Fig. 11*b*). This cycle may be interrupted by increasing natural visual stimulation outdoors (Poudel et al., 2023).

It should be noted that low peripheral contrast has been recently used to suppress ∼0.4 diopters of myopia progression in children (Rappon et al., 2023). Although more work is needed to confirm and interpret this result (Breher et al., 2023), low-contrast environments could potentially protect against myopia by increasing the ON/OFF pathway stimulation balance in favor of the ON pathway. Because the ON pathway responds stronger to lower contrasts than the OFF pathway, low-contrast images should reduce the visual responses of OFF more than ON pathways and make light stimuli more visible than dark stimuli (Rahimi-Nasrabadi et al., 2023). Far vision outdoors also reduces image contrast and should bias the ON/OFF stimulation balance toward the ON pathway by increasing both exposure to low light contrast and bright surfaces. However, because myopia subjects have reduced contrast sensitivity, they may be less inclined to spending time looking at low-contrast targets at far distance.

To summarize, there is strong evidence that poor stimulation of ON pathways drives myopia progression, the most extreme example being monocular visual deprivation (Wiesel and Raviola, 1977). At the same time, weak ON pathways can lead to visual behaviors that reduce ON pathway stimulation (e.g., deficits in pupil constriction can make subjects avoid exposure to bright surfaces outdoors). Therefore, the relation between ON pathway activation and myopia progression may be best described by a circular feedback that relates ON pathway function with visual behavior (Fig. 11).

## Discussion

Our results demonstrate pronounced differences in the contrast response functions of ON and OFF retinal pathways in humans, which closely match differences previously reported in the retina, thalamus, and visual cortex of macaques, cats, guinea pigs, and rats (Chichilnisky and Kalmar, 2002; Zaghloul et al., 2003; Kremkow et al., 2014; Pons et al., 2017; Ravi et al., 2018; Archer et al., 2021; Rahimi-Nasrabadi et al., 2021). As in human visual cortex (Rahimi-Nasrabadi et al., 2021), we demonstrate that contrast sensitivity is higher in ON than that in OFF retinal pathways and the difference increases with luminance range. Moreover, we demonstrate that, in myopia, ON pathways are weaker, slower, less sensitive, and less effective at constricting the pupil and protect the retina from bright light. We have previously demonstrated that low contrasts are more common among light than those among dark stimuli in outdoor scenes, which may explain why ON pathways have higher contrast sensitivity than OFF pathways (Rahimi-Nasrabadi et al., 2021). Taken together with previous work, our results indicate that poor stimulation of ON pathways in low light environments may lead to ON pathway deficits and myopia progression (Jones et al., 2007; Rose et al., 2008; Ashby et al., 2009; Pons et al., 2017, 2019; Poudel et al., 2023).

### Differences in contrast sensitivity between ON and OFF visual pathways

ON pathways have higher contrast sensitivity than OFF pathways in different species (Chichilnisky and Kalmar, 2002; Zaghloul et al., 2003; Kremkow et al., 2014; Pons et al., 2017; Ravi et al., 2018; Archer et al., 2021; Rahimi-Nasrabadi et al., 2021), and this ON–OFF sensitivity difference changes with luminance range (Rahimi-Nasrabadi et al., 2021). When using mid-gray backgrounds in standard monitors with low luminance range (∼100–200 cd/m^2^), visual stimuli drive weaker cortical responses from ON than OFF cortical pathways, and the ON–OFF sensitivity differences are small (Kremkow et al., 2014; Pons et al., 2017; Rahimi-Nasrabadi et al., 2021). However, when the luminance range is increased with high luminance monitors or by changing the background luminance in standard monitors, the responses from ON and OFF cortical pathways become stronger and the ON–OFF sensitivity differences larger (Kremkow et al., 2014; Rahimi-Nasrabadi et al., 2021). Only when the luminance range is kept constant, the strength and sensitivity of the cortical responses remain constant even if the background luminance changes (Rahimi-Nasrabadi et al., 2021).

As in the visual cortex, our results in human retina demonstrate that increasing the luminance range enhances the ON–OFF pathway differences in contrast sensitivity. However, unlike in the visual cortex, the ON–OFF differences measured with flash electroretinography change with both luminance range and background luminance. Increasing the luminance range from 250 to 500 cd/m^2^ makes responses stronger in OFF than that in ON retinal pathways, whereas changes in background luminance makes ON pathway responses on dark backgrounds stronger than OFF pathway responses on bright backgrounds. Therefore, combined changes in luminance range and background luminance can cause pronounced fluctuations in the ON/OFF response ratio measured with flash electroretinography, from strong OFF dominance to strong ON dominance. Conversely, in the visual cortex, the ON/OFF response ratio remains nearly constant under different conditions of background luminance as long as the luminance range remains also constant (Rahimi-Nasrabadi et al., 2021).

### ON–OFF sensitivity differences decrease from the retina to visual cortex

The different effect of background luminance on retinal and cortical responses is likely to reflect different stages of visual processing. The flash electroretinogram is generated by retinal interneurons at the earliest processing stages, just one synapse away from the photoreceptor. Conversely, the cortical encephalogram is generated by neurons that are multiple synapses apart from the retina. Our cortical measurements from both humans and cats are driven by central vision whereas the flash electroretinogram is generated by the entire retina. However, visual eccentricity is unlikely to explain the retina–cortical response differences because changes in background luminance also affect the ON–OFF response ratio measured with pattern electroretinography in the central retina.

The contrast response functions of ON and OFF pathways measured with electroretinography in humans closely replicate measurements from single retinal ganglion cells in isolated retinas of macaques and guinea pigs (Chichilnisky and Kalmar, 2002; Zaghloul et al., 2003; Ravi et al., 2018). The similarity between the contrast response functions of human and guinea pigs is particularly striking (Zaghloul et al., 2003; see their Fig. 2*C* right panel with our Fig. 3). In both humans and guinea pigs, the response to high contrasts is stronger in OFF than that in ON pathways, whereas the response to low contrasts is stronger in ON than that in OFF pathways, consistently with measurements in the visual cortex (Rahimi-Nasrabadi et al., 2021 and human vision Rahimi-Nasrabadi et al., 2023). Moreover, in both humans and guinea pigs, the OFF pathway responds poorly to contrasts lower than 20%, which makes the ON/OFF sensitivity ratios larger in the retina than that in the cortex. For example, the average OFF pathway C50n was 0.61 in our human retinal measurements and 0.6 in the retinal measurements from guinea pigs reported by Zaghloul et al. (2003; Fig. 2*C*, right panel). By comparison, the average OFF pathway C50n in the visual cortex was 0.37 in humans and 0.42–0.46 in cats (0.42 at 300 cd/m^2^ and 0.46 at 1,000 cd/m^2^ luminance range; data from Rahimi-Nasrabadi et al., 2021).

The differences in contrast sensitivity between ON and OFF visual pathways are also present in human vision. At low 5% contrast, humans make more errors and are slower at detecting dark than light targets but as the contrast increases, they make more errors and are slower at detecting light than dark targets (Rahimi-Nasrabadi et al., 2023). The differences in ON–OFF contrast sensitivity are smaller in visual perception than those in the retina and visual cortex. For example, at a photopic luminance range of ≤500 cd/m^2^, the OFF/ON C50n ratio is 1.5–2 in the retina (C50n OFF/ON, 0.61/0.4 in humans and 0.6/0.3 in guinea pigs; data from Fig. 3*g* of this paper and Fig. 2*c* of Zaghloul et al., 2003), 1.3 in the visual cortex (0.37/0.28 in humans and 0.42/0.32 in cats; data from Supplementary Fig. 3*c* and Fig. 3*c* of Rahimi-Nasrabadi et al., 2021), and close to 1 in human vision (C50n OFF/ON, 0.0656/0.0655; data from Fig. 4*a* of Rahimi-Nasrabadi et al., 2023). Light targets are only detected better than dark targets at very low contrasts (Pons et al., 2017; Rahimi-Nasrabadi et al., 2021), which explains why staircase methods not sampling the lowest contrasts frequently find larger thresholds for light than dark stimuli (Stoimenova, 2007). Contrast sensitivity also increases with the size of the human primary visual cortex (Himmelberg et al., 2022; Jigo et al., 2023). Therefore, taken together, these results indicate that neuronal convergence at successive stages of visual processing makes contrast thresholds more dependent on neuronal density than retinal sensitivity consistent with recent models of cortical topography (Najafian et al., 2022).

### ON retinal pathways are weaker in myopia

Our results demonstrate that myopia makes the electroretinogram weaker but affects differently the ON and OFF pathways. We show that myopia reduces the responses to low–medium contrasts in ON more than OFF pathways, increases the response latency in ON but not OFF pathways, and makes ON pathways slower and less effective at driving pupil constriction. These results may explain why myopia can affect both light sensitivity (Qin et al., 2010) and contrast sensitivity (Jaworski et al., 2006; Stoimenova, 2007; although contrast sensitivity may also decrease with retinal stretching in high myopia).

Our previous work also demonstrated that ON pathways are better driven than OFF pathways by high spatial frequencies (Kremkow et al., 2014; Jansen et al., 2019; Pons et al., 2019), bright surfaces, and luminance transients (Xing et al., 2014; Mazade et al., 2019; Mazade et al., 2022), which are all common in outdoor scenes. Therefore, outdoor activity may protect against myopia progression (Rose et al., 2008; French et al., 2013; Wu et al., 2020) by strongly activating ON visual pathways (Pons et al., 2017; Poudel et al., 2023). Consistent with this hypothesis, dopamine is released in the retina through ON dopaminergic amacrine cells (Dacey, 1990; Munteanu et al., 2018), and dopaminergic agonists prevent myopia progression in different species (Iuvone et al., 1991; Rohrer et al., 1993; Dong et al., 2011; Yan et al., 2015; Zhou et al., 2017). Moreover, complete ON pathway inactivation causes high myopia in humans (Miyake et al., 1986; Boycott et al., 1998; Dryja et al., 2005; Kim et al., 2021) and increases induced myopia progression in mice (Pardue et al., 2008; Chakraborty et al., 2015). Reading black text in white background also reduces ON pathway stimulation (Aleman et al., 2018; Poudel et al., 2023) and increases the risk of myopia progression in humans (Mutti et al., 2002). Moreover, as we demonstrate here, myopia progression is associated with pronounced deficits in ON pathway function that affect the ability of the retina to signal contrast, generate fast responses to luminance transients, and drive pupil constriction. Therefore, taken together with our previous work (Pons et al., 2017; Pons et al., 2019; Poudel et al., 2023), our results support the hypothesis that ON pathway stimulation may prevent myopia progression.
